# Quantitative 3-D morphometric analysis of individual dendritic spines

**DOI:** 10.1038/s41598-018-21753-8

**Published:** 2018-02-23

**Authors:** Subhadip Basu, Punam Kumar Saha, Matylda Roszkowska, Marta Magnowska, Ewa Baczynska, Nirmal Das, Dariusz Plewczynski, Jakub Wlodarczyk

**Affiliations:** 1 Department of Computer Science and Engineering, Jadavpur University, Kolkata, 700032 India; 2 Department of Electrical and Computer Engineering and Department of Radiology, University of Iowa, Iowa City, IA 52242 USA; 3 Nencki Institute of Experimental Biology, Polish Academy of Sciences, Pasteura 3, Warsaw, 02-093 Poland; 4 Center of New Technologies, University of Warsaw, Banacha 2c Street, Warsaw, 02-097 Poland

## Abstract

The observation and analysis of dendritic spines morphological changes poses a major challenge in neuroscience studies. The alterations of their density and/or morphology are indicators of the cellular processes involved in neural plasticity underlying learning and memory, and are symptomatic in neuropsychiatric disorders. Despite ongoing intense investigations in imaging approaches, the relationship between changes in spine morphology and synaptic function is still unknown. The existing quantitative analyses are difficult to perform and require extensive user intervention. Here, we propose a new method for (1) the three-dimensional (3-D) segmentation of dendritic spines using a multi-scale opening approach and (2) define 3-D morphological attributes of individual spines for the effective assessment of their structural plasticity. The method was validated using confocal light microscopy images of dendritic spines from dissociated hippocampal cultures and brain slices (1) to evaluate accuracy relative to manually labeled ground-truth annotations and relative to the state-of-the-art Imaris tool, (2) to analyze reproducibility of user-independence of the segmentation method, and (3) to quantitatively analyze morphological changes in individual spines before and after chemically induced long-term potentiation. The method was monitored and used to precisely describe the morphology of individual spines in real-time using consecutive images of the same dendritic fragment.

## Introduction

Dendritic spines are small membranous extensions on neuronal dendrites that form the postsynaptic site of most of excitatory synapses in the central nervous system. Dendritic spines have distinct structural features and are a heterogeneous group in terms of size and shape^1^. Morphologically, dendritic spines consist of a spine head, where the excitatory synapse is located, which is separated from the parent dendrite by a thin neck that isolates the spine cytoplasm from the dendrite (Harris and Kater, 1994). Such a specific shape allows electrical and biochemical compartmentalization^2–5^. Dendritic spines are essential for the accurate activity and signal transmission of neural circuits, but their exact function is still elusive and remains under intensive investigation^6–8^.

The shape of dendritic spines may undergo activity- and experience-dependent modifications that are believed to associate synaptic plasticity^4,9–12^ with biological phenomena that are critical for synaptic function^13,14^. Although the functional consequences of these morphological changes are not fully understood, the structural and functional plasticity of dendritic spines is widely believed to accompany learning and memory^8^ and many pathological processes e.g. Alzheimer’s disease^15^, Parkinson’s disease^16^. Presently, it is believed that the structural plasticity of dendritic spines is indeed related to synaptic function, since time-dependent morphological dynamics of spines accompany the learning processes^17^. Recent works propose the structural models of synaptic plasticity, linking long-term potentiation with spine enlargement, as opposite to long-term depression, where the synaptic strength weakening is associated with spine shrinkage^18,19^.

Many aspects of the tight structure-function relationship that exists in dendritic spines remain unknown, mainly because of their complex morphology. Whether and the degree to which synaptic strength is modified by structural changes remain unclear^20^. Dendritic spines are unstable structures, and their dynamic nature contributes to existing analytical problems^21^. The limited optical resolution of images obtained using popular confocal microscopy technique, difficulties in dendritic spine segmentation from dendrites, and the identification of true spine boundaries^22–24^ pose challenges to the accurate quantitative analysis of spines in contemporary neurobiology. Progress in imaging technologies allowed the researchers to acquire information about dendritic spines from images obtained both in 2-D and 3-D. Thus, it is possible to examine the complete biological context including interactions with neighboring cells, connectivity, and chemical and protein composition of dendritic spines. The final piece needed to complete the puzzle is a robust and unbiased image analysis tool for quantitative assessment of the information stored in dendritic spines morphology^25,26^.

**Figure 1:**
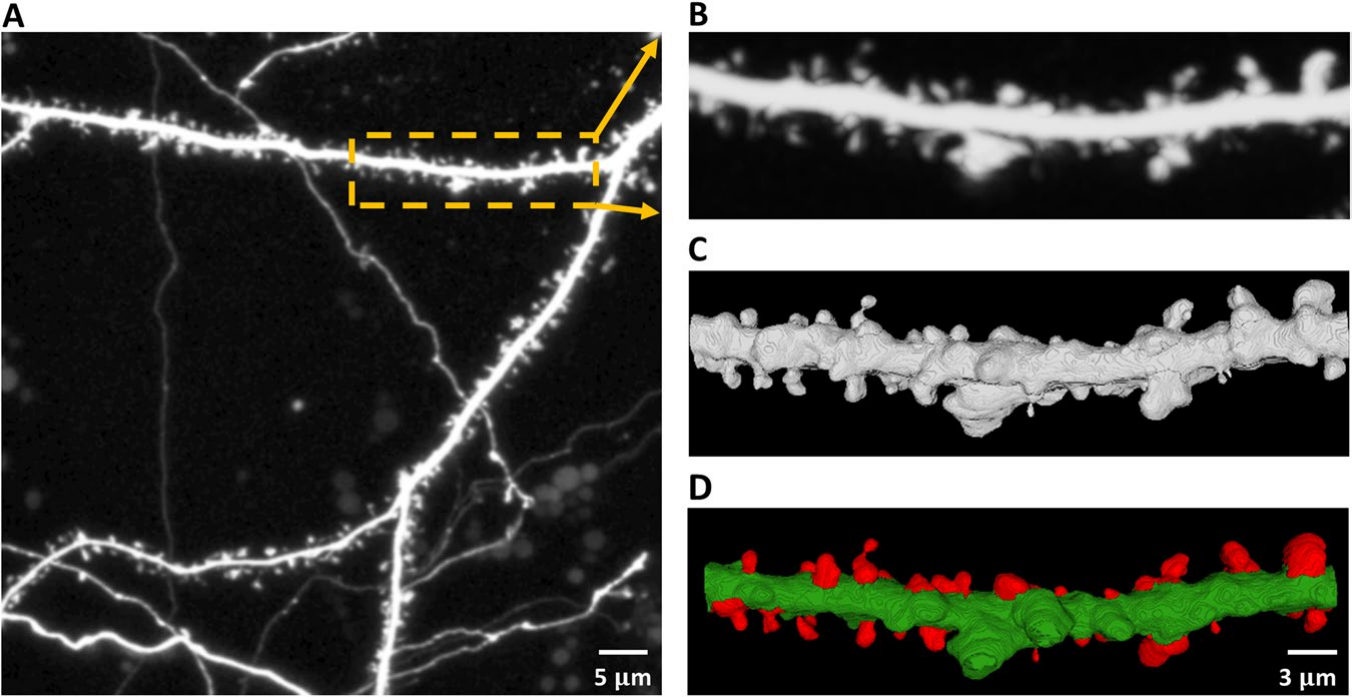
Confocal light microscopy image of hippocampal dendrite covered with dendritic spines. (**A**) Maximum intensity projection (MIP) of the z-stack with an outlined region-of-interest (ROI). (**B**) Cropped and enlarged MIP image of the selected ROI. (**C**) 3-D rendering of the selected ROI from the confocal z-stack with enhanced morphological details of individual spines. (**D**) 3-D segmentation result of the selected ROI with the extracted spines marked in red.

There are predominantly two kinds of approaches when dealing with the analysis of dendritic spines changes. Some methods utilize 2-D MIP (Maximum Intensity Projection) images of dendritic spines providing structural details of individual spines. However, accurate analysis of dendritic spines based on 2-D MIP images is nearly impossible. Therefore, in the present work, we focused on analyzing dendritic spine images based on 3-D volume that were generated from the confocal image stack (see Fig. 1).

A few previous studies addressed the issue of individual spine morphometry. Imaris software^27^ is a commercially available tool for the four-dimensional (4-D) analysis of dendritic spines. Although Imaris software is good for analyzing the overall spine population, it fails to accurately model the three-dimensional (3-D) morphology of individual spines. Swanger *et al*.^28^ also reported an automated method for the 4-D analysis of dendritic spine morphology. However, they used the same Imaris pipeline to develop the automated tool. Both methods generally fail to assess individual spine plasticity.

Among the existing 3-D approaches dealing with morphological analysis of the structure of dendritic spines, Janoos *et al*. proposed a 3-D reconstruction based on skeletonization. In this approach, first the center of the neuron is extracted, then the longest line is considered as the backbone of the dendrite and the shorter lines are considered as the centerlines of dendritic spines^29^. Such a method is time consuming to get the centerlines from the 3-D volume with complex shapes. Additionally, even when images are at the limits of confocal laser scanning microscopy resolution the precision of segmentation is limited due to skeletonization method and by the quantization errors^30^.

A 3-D neuron analysis approach was also proposed by Rodriguez *et al*.^31^. A 3-D reconstruction algorithm uses the Rayburst diameter^31,32^, where Rayburst is defined as casting a multidirectional core of rays from an interior point to the surface of a solid, allowing quantification of anisotropic and irregularly shaped 3D structures. The Rayburst diameter in each layer of a spine is calculated and the head and neck of each spine is defined according to the distribution of the diameters. The aforementioned method detects and classifies the spines in an efficient way, however it may be not accurate in segmentation of very complex structures. The spine shape is defined only by the head to neck ratio and a global threshold of this parameter is not adaptive when performing an analysis of different kind of images^30^.

The latest morphological analysis algorithm for dendritic spines was introduced by Shi *et al*.^30^ and it is based on a semi-supervised learning (SSL) approach. In this framework, first the dendrite backbone is tracked on a 2-D plane then all the dendrites’ surface with meshes is reconstructed in a 3-D. Next, dendritic spines segmentation based on wavelet transform is performed. The segmenting positions are located where the wavelet response on a spine section quickly changes. Features of spines such as the head and neck diameter, spine length, volume, etc. are extracted after the spines segmentation. In the last part of analysis, a small portion of detected spines are selected by a neurobiology expert, and chosen as the training set for classification. The labels of the rest of the spines can be calculated after the training process and all the detected spines can be classified by the learning framework^30^. However the accuracy and performance of the above methods strongly depend on the size of the training dataset and the features included in training vectors.

Here, we propose the fast and accurate methodology which allows studying the morphology of individual spines. Since our method is not based on machine learning approach it does not require the training data set selected by a neurobiologist. We used basic mathematical notations to define different key spine compartments (e.g., spine head and spine neck) and experimentally verified that the quantitative analysis of the newly defined spine attributes accurately modeled spine plasticity. The approach that we developed allows the user to mark specific dendritic spines, segment the spines as 3-D volumes, and extract relevant morphometric features with high accuracy and minimal user intervention.

## Theory and Definitions

### Basic Definitions and Notations

A 3-D cubic grid, or simply a *cubic grid*, is represented by ${Z}^{3}|\,Z$, the set of integers. A *grid point*, often referred to as a *point* or *voxel*, is an element of ${Z}^{3}$ and represented by a triplet of integer coordinates. Standard 26-adjacency^33^ is used here, and two adjacent voxels are often referred to as *neighbors* of each other. The set of 26-neighbors of a voxel *p*, excluding itself, is denoted by *N*(*p*).

An *object* ${\mathscr{O}}$ is a fuzzy subset $\,\{(p,{\mu }_{{\mathscr{O}}}(p))|p\in {Z}^{3}\}$ of ${Z}^{3}$, where ${\mu }_{{\mathscr{O}}}:{Z}^{3}\to [0,1]$ is the membership function. The support *O* of an object ${\mathscr{O}}$ is the set of all voxels with non-zero membership (i.e.,$\,O=\{p|p\in {Z}^{3}\,{\rm{and}}\,{\mu }_{{\mathscr{O}}}(p)\ne 0\}$; $\bar{O}={Z}^{3}-O$ is the *background*). We use a calibrated capital letter to denote a fuzzy subset, whereas a regular capital letter is used to denote its support, which is a binary set. Images are always acquired with a finite field of view. Thus, we will assume that an object always has a bounded support.

Let *S* denote a set of voxels. A *path* $\pi$ in *S* from $p\in S$ to $q\,\in S$ is a sequence 〈$p={p}_{1},{p}_{2},\cdots ,{p}_{l}=q$〉 of voxels in *S* such that every two successive voxels are adjacent. A *link* is a path 〈*p*, *q*〉 of exactly two adjacent voxels. The *length of a path* $\pi =\,\langle {p}_{1},{p}_{2},\cdots ,{p}_{l}\rangle$ in a fuzzy object ${\mathscr{O}}$, denoted as ${{\prod}}_{{\mathscr{O}}}(\pi )$, is the sum of lengths of all links along the path:

1 $${{\prod}}_{{\mathscr{O}}}(\pi )=\sum _{i=1}^{l-1}\frac{1}{2}({\mu }_{{\mathscr{O}}}({p}_{i})+{\mu }_{{\mathscr{O}}}({p}_{i+1}))\parallel {p}_{i}-{p}_{i+1}\parallel ,$$

where $\parallel p-q\parallel$ is the Euclidean distance between *p*, *q*. The *fuzzy distance*^34,35^ between two voxels $p,q\in {Z}^{3}$ in an object ${\mathscr{O}}$, denoted by ${\omega }_{{\mathscr{O}}}(p,q)$, is the length of one of the shortest paths from *p* to *q*:

2 $${\omega }_{{\mathscr{O}}}(p,q)=\mathop{{\rm{\min }}}\limits_{\pi \in {\mathscr{P}}(p,q)}{{\prod}}_{{\mathscr{O}}}(\pi ),$$

where ${\mathscr{P}}(p,q)$ is the set of all paths from *p* to *q*. The *fuzzy distance transform* (FDT) of an object ${\mathscr{O}}$ is an image $\,\{(p,{\Omega }_{{\mathscr{O}}}(p))|\,p\in {Z}^{3}\}$, where ${\Omega }_{{\mathscr{O}}}:{Z}^{3}\to {\Re }^{+}|\,{\Re }^{+}$ is the set of positive real numbers, including zero, that is the fuzzy distance from the background:

3 $${\Omega }_{{\mathscr{O}}}(p)=\mathop{{\rm{\min }}}\limits_{q\in O}{\omega }_{{\mathscr{O}}}(p,q).$$

With regard to spine morphology, the challenges are (1) separating the fuzzy objects ${{\mathscr{O}}}_{{\rm{Spine}}}$ from ${{\mathscr{O}}}_{{\rm{Dendrite}}}$, which are fused at various unknown locations and scales, and (2) morphologically defining spine compartments in the segmented fuzzy objects ${{\mathscr{O}}}_{{\rm{Spine}}}$.

The first challenge is addressed using the MSO algorithm^36^ in two sequential steps. Step 1: segmentation of the combined region ${{\mathscr{O}}}_{{\rm{Spine}}}{\cup }^{}{{\mathscr{O}}}_{{\rm{Dendrite}}}$ from the background: Step 2: separation of ${{\mathscr{O}}}_{{\rm{Spine}}}$ and ${{\mathscr{O}}}_{{\rm{Dendrite}}}$. The first step may be trivially achieved using simple thresholding^37^ and connectivity analysis^38^. Let ${\mathscr{O}}$ be the fuzzy segmentation of the combined region that is obtained in Step 1. All subsequent analyses will be confined to the support $O$ of ${\mathscr{O}}$, which will be the “effective image space.” Let $I:O\to [{I}_{{\rm{\min }}}\,\,,\,{I}_{{\rm{\max }}}]$ be the image intensity function over *O*.

In the second step, segmentation is modeled as the opening of two fuzzy objects that are mutually fused at different unknown regions and scales in the *shared intensity space*, *I*. Here, the main challenge is to determine the local size of the suitable morphological operator that can separate multiple small mutually disconnected structures (e.g., spines) from a large connected structure (e.g., dendrite). The developed MSO algorithm combines FDT^39^ and fuzzy connectivity^40^ to iteratively open the two objects in *I*.

### Multi-scale Opening Algorithm

The basic idea of the MSO algorithm^36^ is to gradually erode the assembly of two fused objects until those two objects become mutually disconnected, thus creating two separate objects. The first iteration starts with two sets of seed voxels, ${S}_{{\rm{Spine}}}$ and ${S}_{{\rm{Dendrite}}}$, and a set of common separators, *S*_*S*_. The initial FDT map ${\Omega }_{{\rm{Spine}},0}$ for the first object is computed from $O$, except that the voxels in ${S}_{{\rm{Dendrite}}}\cup {S}_{S}$ are added to the background. The FDT map ${\Omega }_{{\rm{Dendrite}},0}$ for the other object is computed similarly. It is reasonable to assume that the sets ${S}_{{\rm{Spine}}}$, ${S}_{{\rm{Dendrite}}}$, and *S*_*S*_ are mutually exclusive.

**Figure 2:**
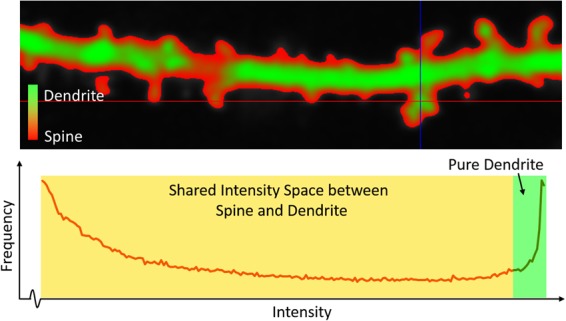
Intensity distribution between the dendrite and spine segments in a sample confocal light microscopy image. (Top) Color-coded regions in a dendritic segment. (Bottom) Intensity histogram of the dendritic segment that represents the overlap of the pure dendrite region and shared space between the dendrite and spines.

Let us now consider the coupling of two objects, where a dendritic segment (green) and the spines (red), with significant intensity overlap (illustrated in Fig. 2), are fused with each other at different unknown locations and scales. Let ${{\rm{\mu }}}_{{\rm{Dendrite}}}$ and ${{\rm{\mu }}}_{{\rm{Spine}}}$ denote the dendrite and spine membership functions, defined as the following:

4 $${\mu }_{{\rm{Dendrite}}}(p)=\{\begin{array}{ll}0, & {\rm{if}}\,I(p) < {I}_{{\rm{Spine}}},\\ \frac{I({\rm{p}})-{I}_{{\rm{Spine}}}}{{I}_{{\rm{Dendrite}}}-{I}_{{\rm{Spine}}}}, & {\rm{if}}\,{I}_{{\rm{Spine}}}\le I(p) < {I}_{{\rm{Dendrite}}},\\ 1 & {\rm{otherwise}},\end{array}$$

5 $${\mu }_{{\rm{Spine}}}(p)=\{\begin{array}{l}\begin{array}{cc}0, & \,\mathrm{if}\,I(p)\end{array} < {I}_{{\rm{\min }}},\\ \begin{array}{cc}1, & {\rm{if}}\,{I}_{{\rm{\min }}}\le I(p)\end{array} < {I}_{{\rm{Spine}}},\\ \begin{array}{cc}\frac{{I}_{{\rm{Dendrite}}}-I(p)}{{I}_{{\rm{Dendrite}}}-{I}_{{\rm{Spine}}}}, & {\rm{if}}\,{I}_{{\rm{Spine}}}\le I(p) < {I}_{{\rm{Dendrite}}},\,\end{array}\\ \begin{array}{cc}0, & \,\mathrm{if}\,I(p)\,\ge \end{array}\,{I}_{{\rm{Dendrite}}},\end{array}$$

where $I:\,O\to [{I}_{{\rm{\min }}}\,\,,\,{I}_{{\rm{\max }}}]$ is the image intensity function over $O$. ${I}_{{\rm{Spine}}}$, and ${I}_{{\rm{Dendrite}}}$ is the representative spine and dendrite intensities that define the respective transition between pure and shared intensity bands (see Fig. 2). Let ${P}_{{\rm{Dendrite}}}\subset O$ and ${P}_{{\rm{Spine}}}\subset O$ be the set of voxels that fall inside the pure intensity band for dendrite and spine respectively. Thus, the set of voxels that fall within the shared intensity band is ${O}_{{\rm{Shared}}}=O-{P}_{{\rm{Dendrite}}}-{P}_{{\rm{Spine}}}$. A fuzzy representation of the composite object may be obtained by taking the fuzzy union of the two membership functions that are shown in Equations 4 and 5. The iterative approach of the multi-scale opening of two structures takes several iterations to grow the path-continuity of an object, starting from its seed voxels (commonly added in large-scale regions), to a peripheral location with fine-scale details.

Note that after the iterative propagation of the MSO algorithm, the dendrite region is segmented as a single connected component. $\,{O}_{{\rm{Spine}}}$ represents one or more disjointed spine regions (${R}_{i}$), such that $\,{O}_{{\rm{Spine}}}={\cup }_{i=1}^{K}{R}_{i}$, where $K$ is the total number of disjointed spine segments in $\,{O}_{{\rm{Spine}}}$, and each such segmented spine region ${R}_{i}$ contains at least one spine seed $p\in {S}_{{\rm{Spine}}}$.

### Morphological definitions for the spine regions

Once the spines are segmented from the dendrite, the challenge is to assess the morphological attributes accurately. The morphology of the dendritic spines is complex and difficult to quantify. In most cases, they are described by simple parameters, such as *length* and *head width*^41^, in 2-D MIP images of the z-stacks that are acquired from the confocal images of dissociated hippocampal cultures. Previously, we developed a convolution kernel-based approach for the segmentation of spines from 2-D MIP images^42^. However, high-resolution confocal microscopy and effective 3-D rendering allow the visualization of complex structures in greater detail.

In the present study, we defined several key morphological features of 3-D dendritic spines for plasticity analysis. Specifically, we defined four key spine features that are related to the base and head of a spine using standard notations of digital topology and geometry^43,44^.

#### *Definition* 1.

For a given spine ${R}_{i}\subset \,{O}_{{\rm{Spine}}}$, the *base* of the spine is defined as the set of points ${B}_{i}\subset {O}_{{\rm{Dendrite}}}$ such that $\forall \,p\in {B}_{i}$, $\exists \,q\in {R}_{i}$ is adjacent to $p$.

#### *Definition* 2.

For a given spine ${R}_{i}\subset {O}_{{\rm{Spine}}}$, the *central base point* $CB{P}_{i}$ is the centroid of the base of the spine ${R}_{i}$ (i.e., $CB{P}_{i}=\frac{1}{|{B}_{i}|}\sum _{\forall \,p\in {B}_{i}}\,p$, where $|\cdot |$ is the cardinality of a set).

The head and tip of a spine are defined using the FDT map^43,44^ ${{\rm{\Omega }}}_{i}$ of ${R}_{i}$. A locally deepest point in a spine ${R}_{i}$ is a point $p\in {R}_{i}$ such that $\forall \,q\in {{\mathscr{N}}}_{l}(p)$ ${{\rm{\Omega }}}_{i}(q)\le {{\rm{\Omega }}}_{i}(p)$, where ${{\mathscr{N}}}_{l}(p)$ is the ${(2l+1)}^{3}$ neighborhood of $p$. Here, $l=2$ is used to avoid noisy local maxima.

#### *Definition* 3.

The *center of the head* $C{H}_{i}$ of a spine ${R}_{i}$ is the locally deepest point in the spine. In a situation where multiple locally deepest points satisfy the farthest distance criterion, their centroid is used.

#### *Definition* 4.

The *tip of a spine* ${T}_{i}$ of a spine ${R}_{i}$ is a point ${T}_{i}\in {R}_{i}$ that is farthest from its central base point $CB{P}_{i}$. In a situation where multiple points of ${R}_{i}$ satisfy the farthest distance criterion, their centroid is used.

**Figure 3:**
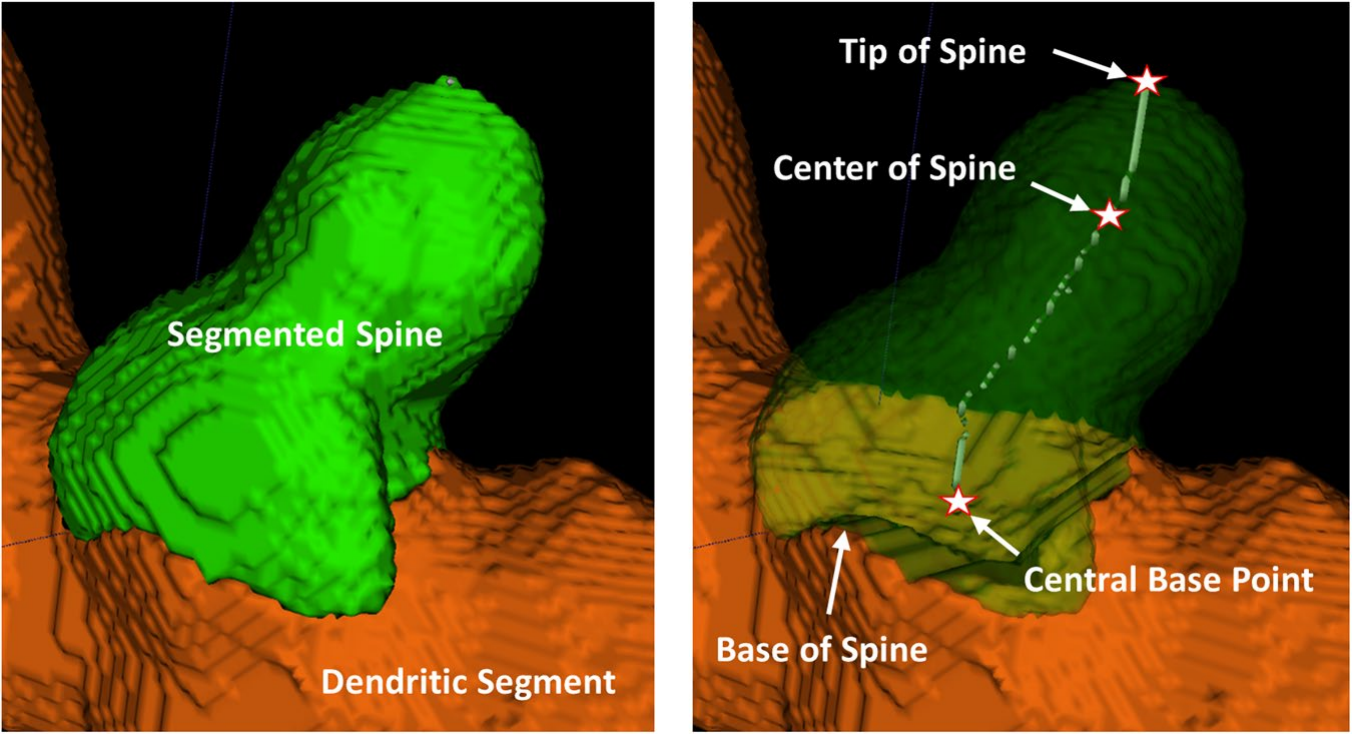
Illustration of a segmented spine structure with automatic quantitative assessment of different spine attributes.

Note that $CB{P}_{i}$, $C{H}_{i}$, and ${T}_{i}$ play key roles in estimating spine attributes, such as *length of the spine*, *neck-length*, *neck-width*, *head-width*, etc., for each individual spine ${R}_{i}$. To estimate these features, we further extended the above definitions to find the geodesic path from *base to head* $B{H}_{i}$ of the spine ${R}_{i}$ by joining the two central points $CB{P}_{i}$ and $C{H}_{i}$ such that $\sum _{\forall \,p\in B{H}_{i}}{{\rm{\Omega }}}_{i}(p)$ is minimized. Likewise, we computed the central path from *head to spine-tip* $H{T}_{i}$ of the spine ${R}_{i}$ by joining $C{H}_{i}$ and ${T}_{i}$, such that $\sum _{\forall \,p\in H{T}_{i}}{{\rm{\Omega }}}_{i}(p)$ is minimized. Figure 3 shows an illustration of these key spine attributes with respect to a segmented spine.

We now estimate the *neck length* $N{L}_{i}$ of ${R}_{i}$ as $N{L}_{i}=B{H}_{i}-{{\rm{\Omega }}}_{i}(C{H}_{i})$. *Minimum neck-width* $MN{W}_{i}$ of ${R}_{i}$ is estimated as $MN{W}_{i}={\min }_{\forall p\in B{H}_{i}}({{\rm{\Omega }}}_{i}(p)).$ *Average head-width* $AH{W}_{i}$ of the spine ${R}_{i}$ is estimated as $AH{W}_{i}=$ ${{\rm{avg}}}_{\forall p\in H{P}_{i}}({{\rm{\Omega }}}_{i}(p))$ such that $H{P}_{i}$ is the set of all locally deepest points in ${R}_{i}$. Finally, the *length of the spine* ${L}_{i}$ is estimated as ${L}_{i}=|B{H}_{i}|+|H{T}_{i}|$.

**Figure 4:**
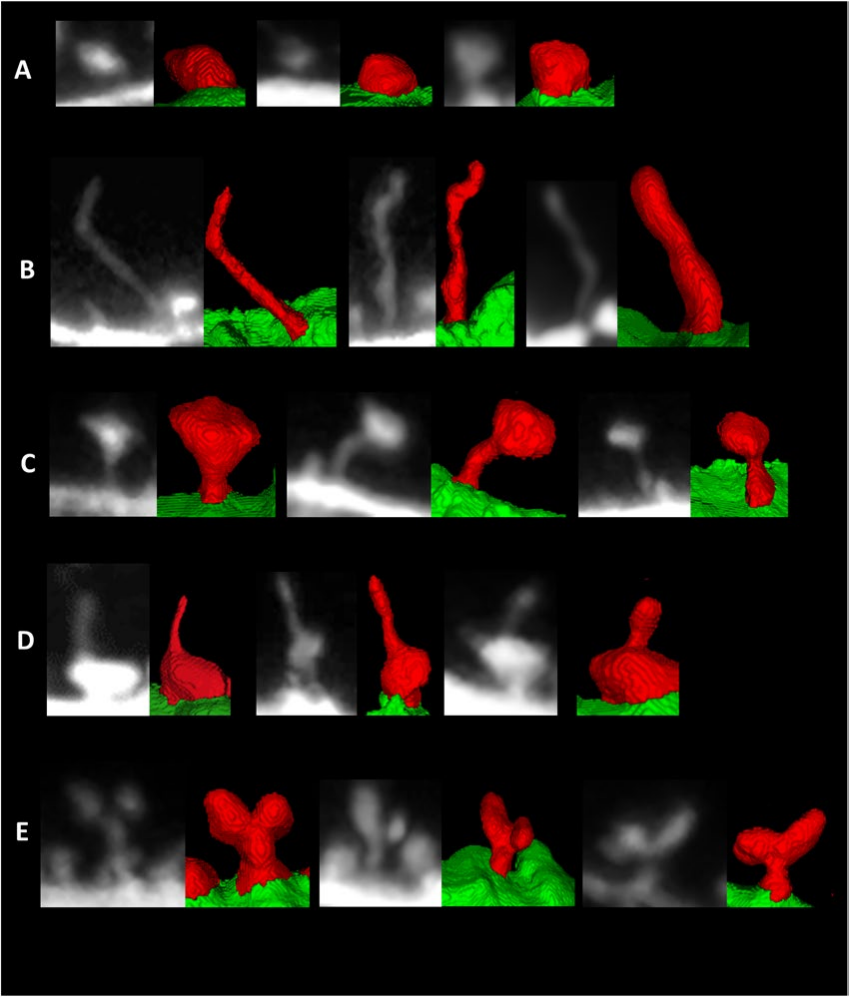
Examples of the segmented spines of different categories that were obtained by the currently developed method. (**A**) stubby, (**B**) filopodia, (**C**) mushroom, and (**D**) spine-head protrusions, (**E**) branched spines.

Using these morphological definitions, we classify the spine shapes into one of the four categories: Stubby, Mushroom, Filopodia and Spine-head Protrusion. Formal morphological definitions for these spine categories are discussed by Basu *et al*.^42^. The basic classification logic relies on the accurate estimation of the *neck length* $(N{L}_{i})$ of a spine. If the *neck length* is zero, we classify a spine as Stubby. Otherwise, we check the locally deepest points $(C{H}_{i})$ in each spine. In case of a thin Filopodia, the locally deepest points are usually spread along the length $({L}_{i})$ of the spine. This is not common in Mushroom or Spine-head Protrusions, where the locally deepest points are usually concentrated around the *spine-head* regions. We utilize this attribute to identify the Filopodia type of spines. Then, to classify the Mushroom type spines we estimate the *base-to-head* distance $(B{H}_{i})$ of a spine. We have observed that the Mushroom spines have short *base-to-head* distance in comparison to the overall *length of spine*. Finally, the ratio $B{H}_{i}/{L}_{i}$ decisively classifies the Mushroom spines from the Spine-head Protrusions. Spine classification results on different dendritic segments are shown in Fig. 4. Apart from the four spine categories we have also observed some branched or bifurcated spines (see Fig. 4E), which could not be decisively classified into any of the four classes. Although they are few in numbers, we have placed them in a separate category for future analysis.

## Experimental Results

The methodology that we developed is useful in a variety of applications that involve the accurate volumetric assessment of spine plasticity. Two specific challenges are involved in this process: (1) accurate 3-D segmentation of individual spines from the dendritic segment and (2) quantitative analysis of individual spine morphology for the effective assessment of structural changes in dendritic spines. Specifically, we analyzed spine plasticity using the 3-D morphological features that are presented in the previous section. The confocal light microscopy images of dendritic spines from dissociated hippocampal cultures were used for (1) the analysis of accuracy relative to ground-truth annotations that were generated by experimental biologists and the available state-of-the-art Imaris tool, 2) the analysis of reproducibility of user-independence of the segmentation results, and (3) the quantitative analysis of morphological changes in spines. In the following section, we describe the image acquisition protocol and image sets that were used for experimental validation. We then present the accuracy analysis of the currently developed method relative to ground-truth annotations and comparisons with the state-of-the-art Imaris tool. We also present the reproducibility analysis using three mutually blinded experts. Finally, we discuss structural changes in spines.

### Description of the datasets

This study was carried out in accordance with the Ethical Committee on Animal Research of the Nencki Institute, based on the Polish Act on Animal Welfare and other national laws that are in full agreement with EU directive on animal experimentation. All effort was made to minimize animal suffering. Dissociated hippocampal cultures from postnatal day 0 Wistar rats were prepared as described in Basu *et al*.^42^. Cultured hippocampal neurons were transfected 14 days *in vitro* (DIV) with Syn-GFP plasmid to visualize neuronal morphology. Live-cell imaging was performed on 20–22 DIV. Dendritic segments that were decorated with dendritic spines were imaged at time 0, before stimulation, and then cLTP was induced by bath application of a mixture of 50 μM forskolin, 50 μM picrotoxin, and 0.1 μM rolipram (each dissolved in dimethylsulfoxide [DMSO]) in maintenance media. Dendritic segments were imaged 10 min and 40 min after cLTP induction. Images were acquired using a Carl Zeiss LSM780 confocal microscope with a C-Apochromat 40×/1.2 NA water immersion objective using a 488 nm wavelength argon laser at 3% transmission and 70 nm/pixel resolution. A series of z-stacks were acquired at 0.2 μm steps.

In the first dataset, three different neurons from rat dissociated hippocampal cultures were imaged using a confocal light microscope, before and after cLTP induction. All of the images were captured three times: at baseline (before cLTP) and 10 and 40 min after cLTP induction. In the second dataset, three different neurons from rat dissociated hippocampal cultures were similarly imaged at baseline and 10 and 40 min after mock cLTP induction (i.e., only the solvent, DMSO, was used). During image pre-processing, we took the confocal z-stack and performed Gaussian de-noising on the 3-D image stack. The pre-processed images at time 0 are labeled as T0, and the images that were captured at 10 and 40 min are labeled as T10 and T40, respectively.

The preparation of the brain slices was based on Magnowska *et al*.^45^. For the visualization of changes in the shape of dendritic spines, of 1,1′-Dioctadecyl-3,3,3′,3′-Tetramethylindocarbocyanine Perchlorate (DiI) staining in stressed and control mice were performed. The mice were anesthetized and the transcardinal perfusion with 1,5% paraformaldehyde was performed. Then the brains were dissected and sliced using vibratome. Slices (140 μm thick) that contained the different brain structures recovered for at least 1.5 h at RT. Random dendrite labeling was performed using 1.6 μm tungsten particles (Bio-Rad, Hercules, CA, USA) coated with propelled lipophilic fluorescent dye (DiI; Invitrogen) that were delivered to the cells by gene gun (Bio-Rad) bombardment. Images of dendrites were acquired under 561 nm fluorescent illumination using a confocal microscope (63×objective, 1.4 NA) at a pixel resolution of 1024 × 1024 with a 3.43 zoom, resulting in a 0.07 μm pixel size. A series of z-stacks were acquired at 0.2 μm steps.

### Accuracy Analysis

For accuracy analysis, we first generated ground-truth spine segmentation results for all of the neuronal images by manually labeling ideal spine regions of a sample spine population with the help of experimental biologists using the open-source image processing software Fiji^46^ and ITKSnap^47^. Although both Fiji and ITKSnap are general-purpose image analysis tools, neither of them are capable of extracting morphological attributes that are specific to a dendritic spine.

**Figure 5:**
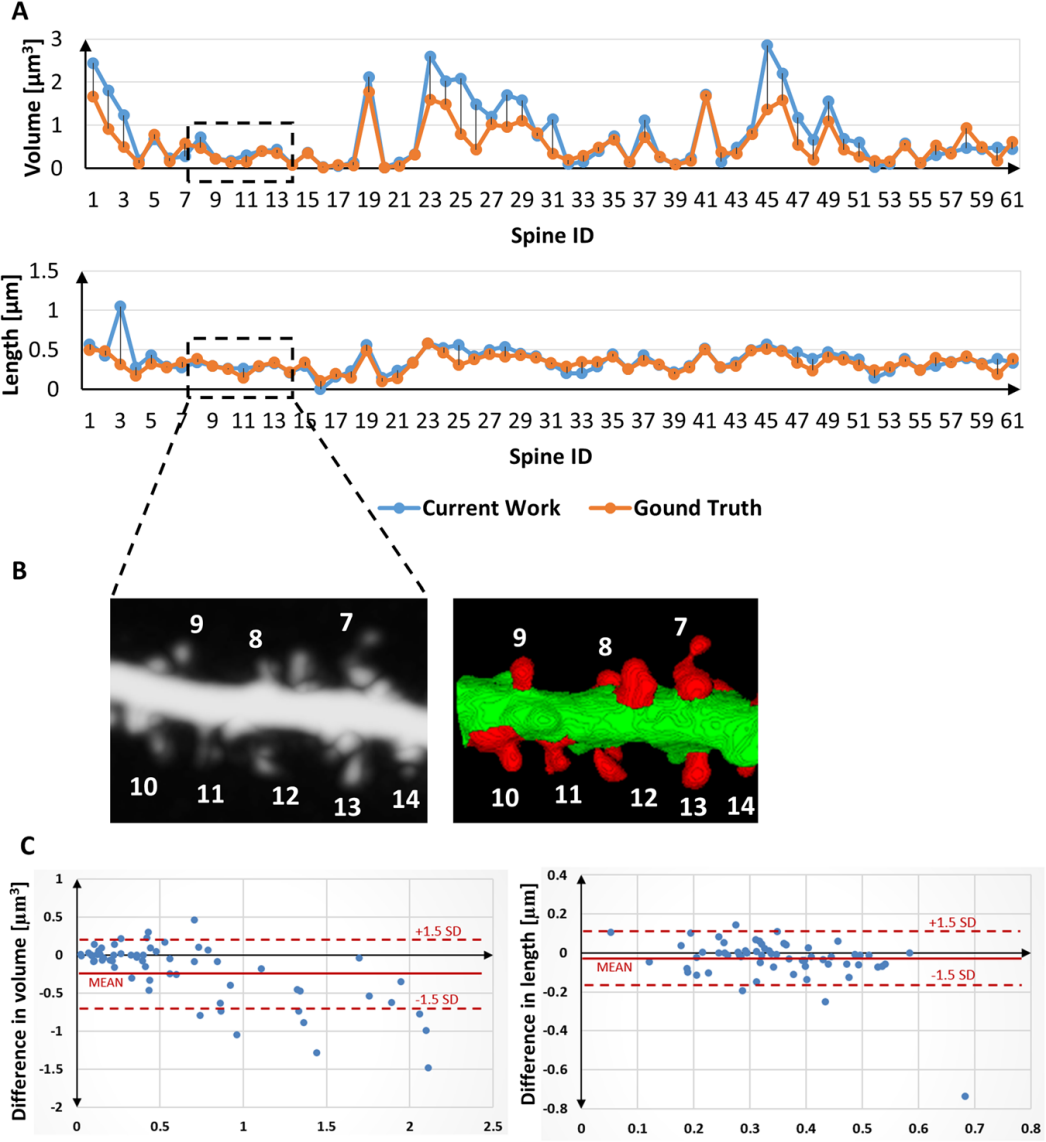
Accuracy analysis of the developed method relative to the manually segmented ground-truth annotations. (**A**) Two standard features (i.e., *volume* and *length*) were considered for the analysis of agreement of the estimated feature values over the sample spine population. (**B**) Cropped image (MIP and 3-D segmentation result) of dendritic segment with numbered spines corresponding to the selected ROI at the graph. (**C**) Difference plots between the estimated by current method and ground-truth values for the spine volume (left) and spine length (right) features (Bland-Altman plots).

For the quantitative analysis of spine segmentation accuracy, we considered the most generic features, such as the *volume* and *length* of a spine. Comparative assessment was performed with regard to the ground-truth annotations that were performed by experts in this domain. The agreement in the estimated feature values and error difference over a sample spine population are illustrated in Fig. 5A. We calculated Pearson’s correlation coefficients to assess the mean agreement of the estimated feature values between the currently developed method and the ground-truth annotations. The respective Pearson’s correlation coefficients for *volume* and *length*, estimated over a sample spine population with manually annotated ground-truth results, were 0.89 and 0.82, respectively. Bland-Altman plots (difference plots) between the estimated values and ground-truth annotations for the two features are shown in Fig. 5C. This is a method of data plotting that is used to analyze agreement between two different data series. Most of the data samples fell within the $\mu \pm 1.5\sigma$ range, representing a significant correlation among the segmented results and ground-truth annotations.

**Figure 6:**
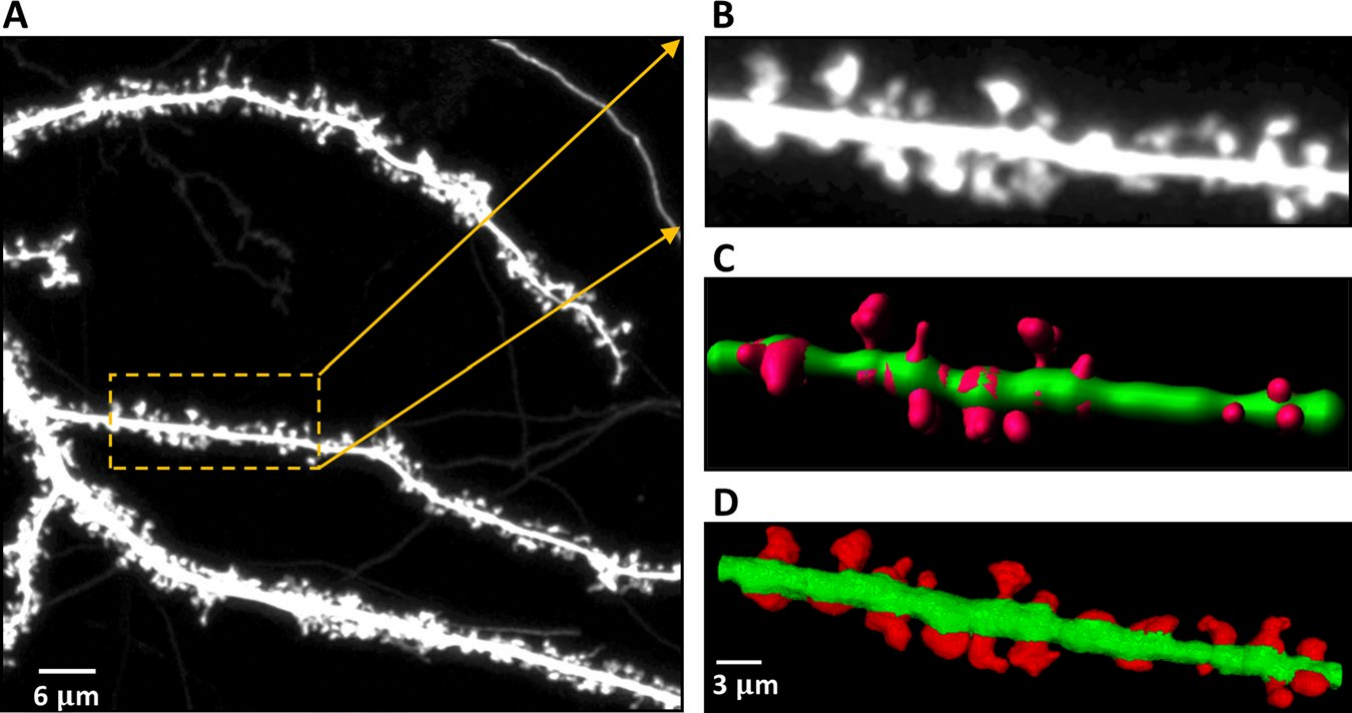
Comparative analysis of the 3-D segmentation results relative to the state-of-the-art Imaris tool^27^ on three sample dendritic segments taken from three different cell images at baseline condition. (**A**) Sample 2-D MIP image with a highlighted ROI segment, (**B**) 2-D MIP image of the selected dendritic segment; (**C**) 3-D segmentation result of image b using the Imaris tool (**D**) 3-D segmentation result of image b using the developed segmentation methodology.

For qualitative comparisons of the 3-D segmentation results, we considered the state-of-the-art Imaris tool^27^ that was applied over the same set of images that were considered in this study. Figure 6 shows the qualitative comparisons of our segmentation results with Imaris. Note that Imaris is a model-based segmentation and feature-extraction tool (outside in) that can lead to the corruption of data. Our proposed method utilizes segmentation-based modeling and feature extraction methodology (inside out). Therefore, the segmentation results and features that are derived are more accurate and robust when considering user variations. Moreover, the spines that are segmented using the currently developed software can be automatically segmented into one of four categories: stubby, filopodia, mushroom, spine head protrusions (Fig. 4) which represent morphological categorization scheme used in EM literature^48–50^. However, the proposed algorithm do not classify the spines as thin because this is an intermediate form of spine^51^ and the mathematical definition of such spine topology does not exist.

Notably, unlike other approaches, our method distinctively identifies spine-head protrusions (Fig. 4D). Moreover, there is another type of spines that we have noted during the segmentation process, namely branched spines (see Fig. 4E). This type of spines are rare and therefore we do not classify them automatically using our present approach, but propose to include in our future work.

### Reproducibility Analysis

**Figure 7:**
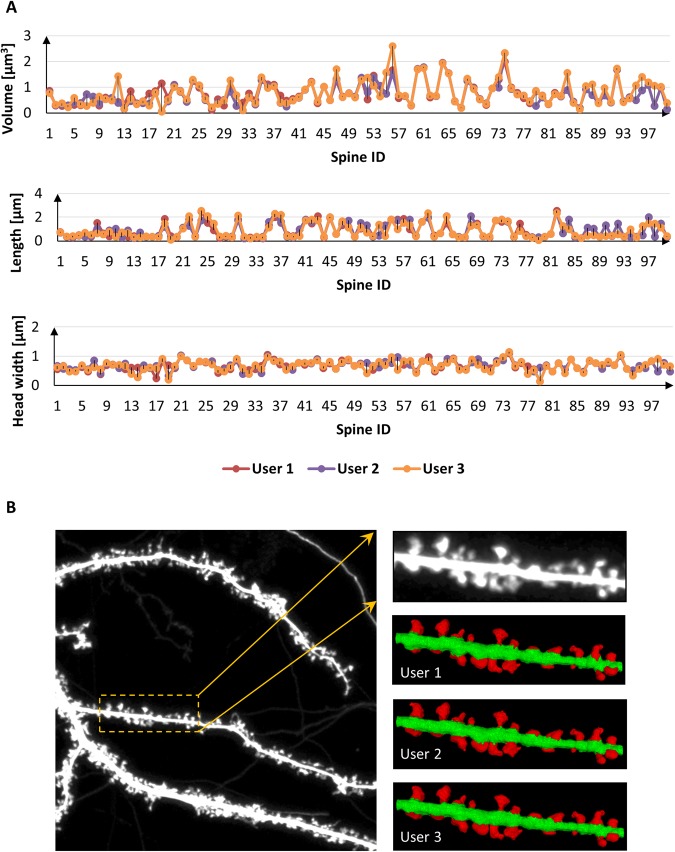
Reproducibility analysis of the currently developed method over a sample spine population with three blinded, independent experts. (**A**) Quantitative analysis of dendritic spine Volume, Length and Head Width performed by three independent users together with (**B**) corresponding 3-D reconstruction results.

In this section, we present multi-user reproducibility of the currently developed method to assess the reliability and robustness of the segmentation methodology. The reproducibility analysis was performed on a sample spine population with three mutually blinded, independent experimental biologists. Figure 7 shows the quantitative and qualitative analysis of volume, length and head width estimations over the sample spine population using three mutually blinded users. The percent standard deviations relative to the mean feature estimation for the three independent users were ±$11 \% ,\,\pm 17 \% \,\,$and ±$6 \%$ for *volume*, *length* and *head-width* respectively.

### Analysis of Spine Plasticity

**Figure 8:**
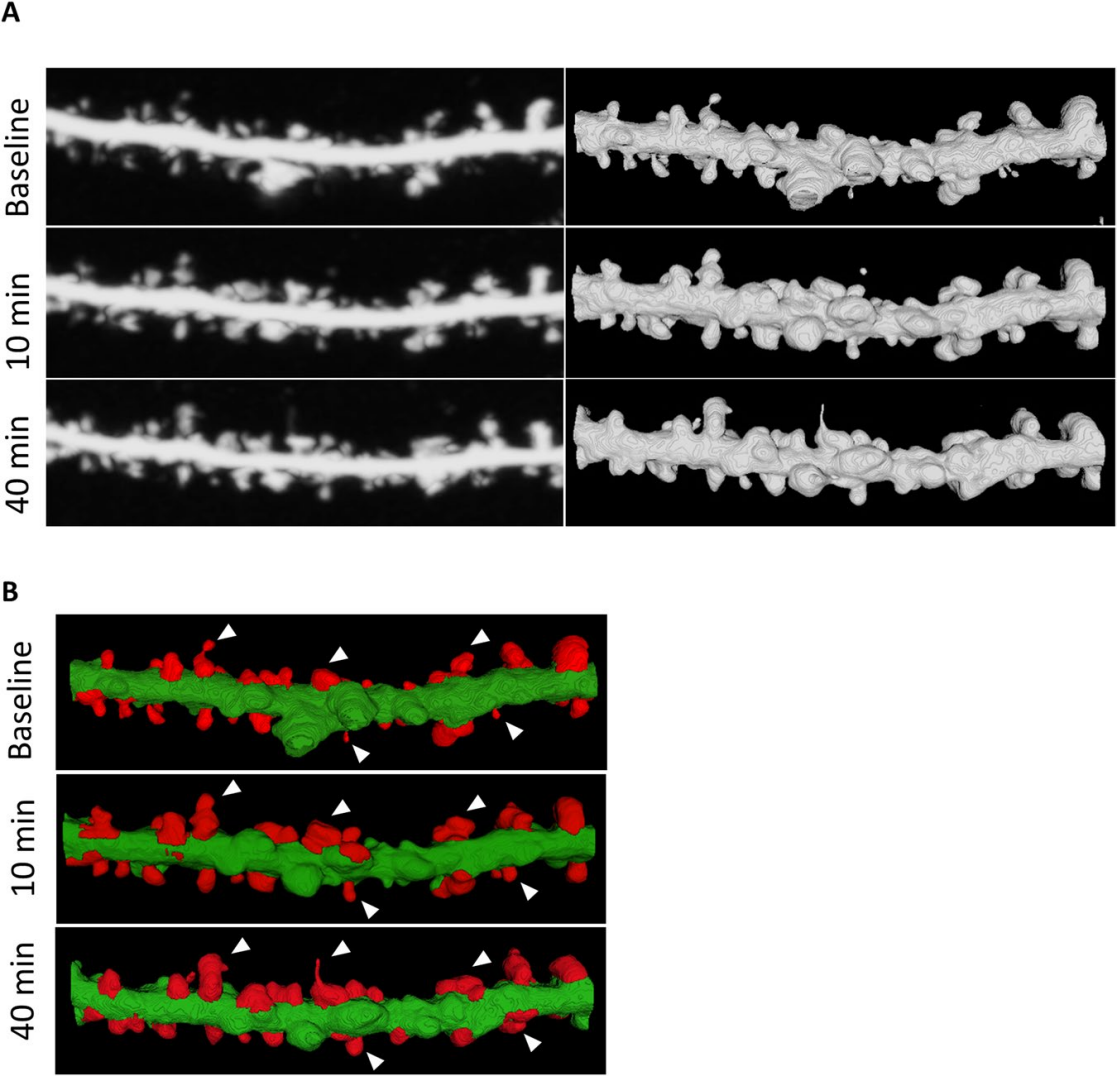
Qualitative assessment of spine plasticity relative to the 3-D segmentation results for a dendritic segment at baseline, 10 minutes and 40 minutes after cLTP induction. (**A**) Comparisons of 3-D rendition results relative to the MIP images, (**B**) 3-D segmented results and morphological changes are observed on sample spines before and after cLTP induction.

**Figure 9:**
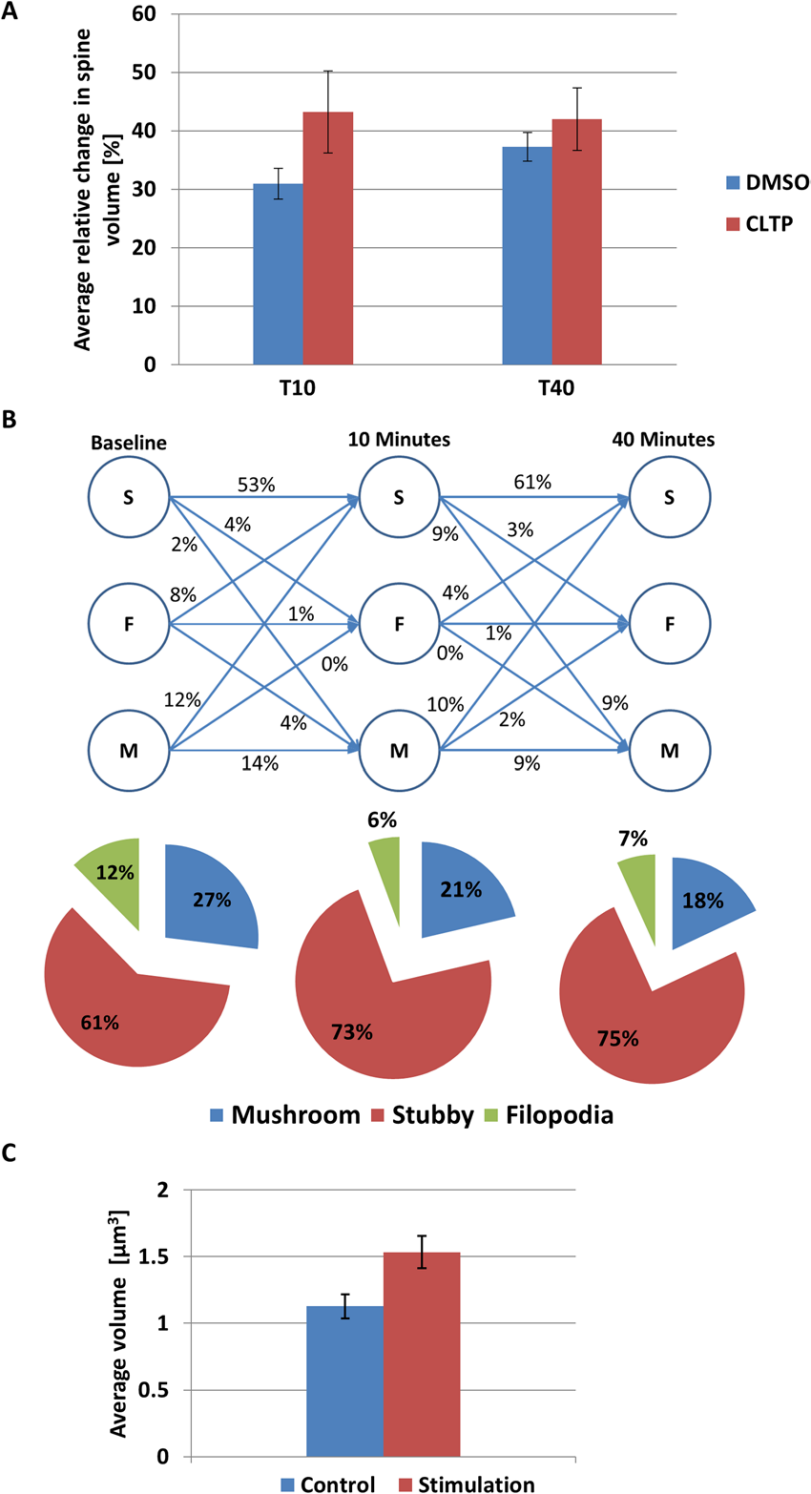
Quantitative analysis of dendritic spine morphology by 3-D segmentation method. (**A**) Average relative changes (percentage) in spine morphology (volume) estimated by the currently developed method at two time points in primary neuronal culture were found to be: 0.94 ± 2.64 (DMSO), 43.25 ± 7.02 (cLTP) at ten minutes stimulation and 37.29 ± 2.43 (DMSO), 42 ± 5.34 (cLTP) at 40 minutes stimulation. (**B**) Changes in dendritic spine morphology (neuronal culture). Results of automatic spine classification where S, F, and M represent spine categories: Stubby, Filopodia and Mushroom respectively. The numbers represent relative transition (in % of total population) between different spine categories before and after 10 minutes and 40 minutes of cLTP induction. (**C**) Average volume of dendritic spines from brain slices before 1.13 ± 0.09 µm^3^ and after stimulation 1.53 ± 0.12 µm^3^. The results are expressed as mean ± SEM.

For detailed qualitative and quantitative analyses of dendritic spines, we assessed the segmentation results of the currently developed methodology over the same dendritic segments that were obtained from different sets of the T0, T10, and T40 images. Figure 8 shows the qualitative assessment of spine plasticity over a sample dendritic segment at the three time points. We present detailed 3-D morphological changes in dendritic spines over time (i.e., before and after cLTP). To investigate the relative changes in morphology of the segmented spines one important feature was considered from the overall experiment. Figure 9A shows the average quantitative changes in dendritic spine volume 10 and 40 min after cLTP induction.

We also show a three-class (stubby, filopodia and mushroom) spine plasticity analysis over three time points (see Fig. 9B). It shows that the 4% the of stubby spines changes to filopodia and 2% changes to mushroom, 8% of the filopodia changes to stubby and 4% changes to mushroom, and 12% of the mushroom changes to stubby from T0 to T10 after cLTP induction. Within the time span of T10 to T40, 3% of the stubby spine changes to filopodia and 9% changes to mushroom, 4% of the filopodia changes to stubby, 10% of the mushroom changes to stubby and 2% changes to filopodia.

**Figure 10:**
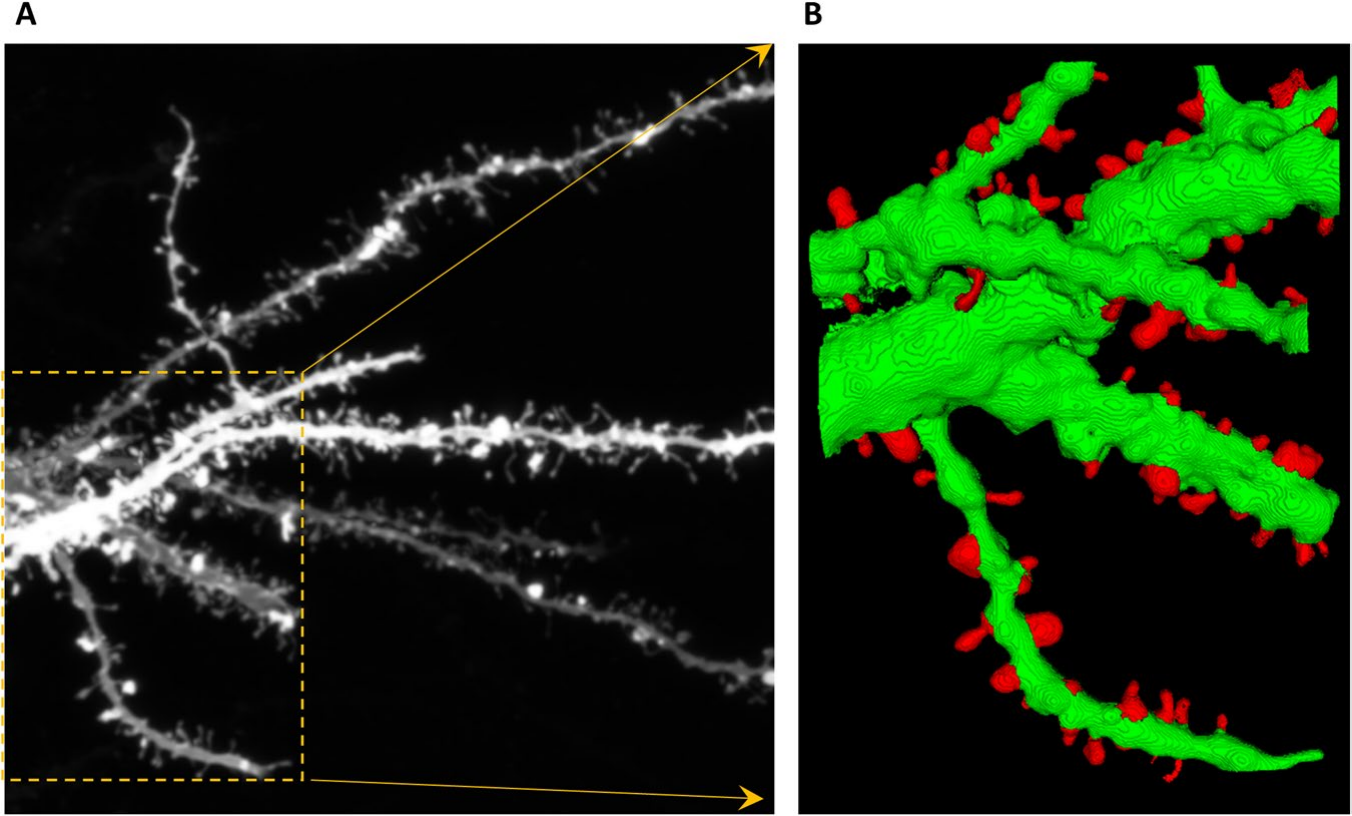
Segmentation of dendritic spines from complex dendritic tree in brain slice. (**A**) 2-D MIP image with a highlighted ROI for 3-D analysis, **(B)** spine segmentation result using the proposed methodology on the selected ROI.

We also investigated the changes in dendritic spines volume from brain slices (Fig. 9C) Sample segmentation results on the images acquired from brain slices are shown in Fig. 10.

We do not provide statistics since our aim is not to assess any particular biological problem, but we aim to show that our method is able to detect differences in spine structure between certain experimental conditions.

## Conclusion and Discussion

There are two critical features related to neuronal signal transmission – connectivity that is related to dendritic spine number and the synaptic strength expressed by dendritic spines structural changes. In the present study we focused on the accurate 3-D segmentation and quantitative assessment of dendritic spine structure. We do not provide the method for dendritic spine density assessment since achieving accurate spine density requires several thousands of spines analyzed, thus it is extremely time consuming to perform with the 3D approach. Analysis of dendritic spine number can be done manually or with the use of 2-D software (e.g. Cheng *et al*.^52^), and such approach is impossible when user wants to get both “real” 3-D spine shape and density.

The segmentation method that we developed was validated for individual spines using real-time experiments and consecutive images of the same dendritic fragment. Our results are consistent with other studies that reported spine head growth upon cLTP induction^45,53^. The plasticity analysis was performed 10 and 40 min after cLTP induction relative to baseline images. The currently developed method was also able to quantitatively assess changes in volume upon stimulation that were consistent with previous studies that reported spine head growth upon cLTP.

Accuracy was evaluated relative to manually labeled ground-truth annotations and relative to the state-of-the-art Imaris tool. We have shown, that the accuracy of the analysis done using Imaris is lower and does not reproduce the exact spine structure and it also fails to capture detailed morphology of individual spines. Imaris program fits only few standard shapes whereas their real multiplicity is much greater. In contrast our method allows to determine the real dendritic spine shape.

To assess the reproducibility of the segmentation results, three blinded experts separately assessed the efficacy of the methodology. The existence of experimental limitations (staining efficiency/quality, image resolution, and detection of fluorescence signal) impedes identification of true spine boundaries whereas our software provides the possibility to minimize user bias.

Although this experiment exclusively used confocal light microscopy images of dendritic spines, the method may be extended in the future for use with other super- resolution imaging techniques, such as photo-activated localization microscopy. The present 3-D segmentation method may be used for different experimental protocols that study the structural dynamics of dendritic spines *in vitro* and *in vivo* under various physiological and pathological conditions. Moreover, we believe that this will be a research tool that enables the detection of even subtle changes in the 3-D dendritic spine structure. Such methodological advances in spine morphological studies will allow more precise analyses and better interpretations of biological data regarding structural plasticity. The present method facilitates accurate, unbiased spine segmentation results and can significantly improve the way we study dendritic spine plasticity. However, it strictly depends on the user knowledge and experimental design which spines are going to be incorporated into the analysis.

### Data Availability

The datasets generated during and/or analyzed during the current study are available from the corresponding authors on reasonable request.
