# Author Correction: Quantitative 3-D morphometric analysis of individual dendritic spines

**DOI:** 10.1038/s41598-018-35164-2

**Published:** 2018-11-15

**Authors:** Subhadip Basu, Punam Kumar Saha, Matylda Roszkowska, Marta Magnowska, Ewa Baczynska, Nirmal Das, Dariusz Plewczynski, Jakub Wlodarczyk

**Affiliations:** 1 Department of Computer Science and Engineering, Jadavpur University, Kolkata, 700032 India; 2 Department of Electrical and Computer Engineering and Department of Radiology, University of Iowa, Iowa City, IA 52242 USA; 3 Nencki Institute of Experimental Biology, Polish Academy of Sciences, Pasteura 3, Warsaw, 02-093 Poland; 4 Center of New Technologies, University of Warsaw, Banacha 2c Street, Warsaw, 02-097 Poland

Correction to: *Scientific Reports* 10.1038/s41598-018-21753-8, published online 23 February 2018

In the original version of this Article, Jakub Wlodarczyk was incorrectly affiliated with “Center of New Technologies, University of Warsaw, Banacha 2c Street, Warsaw, 02-097, Poland”. The correct affiliation is listed below.

Nencki Institute of Experimental Biology, Polish Academy of Sciences, Pasteura 3, Warsaw, 02-093, Poland.

Additionally, the Acknowledgements section was incomplete.

“This work was supported by the National Science Centre Grant - UMO-2015/17/B/NZ3/00557 (J.W., M.R., M.M.) and by an EMMA-West grant from the EU (S. B.). SB was also supported by a FASTTRACK grant (SR/FTP/ETA-04/2012) from DST and a UGC Research Award (F.30-31/2016 SA-II) from the Government of India. D.P. and S.B. were supported by the Polish National Science Centre (2014/15/B/ST6/05082) and D.P. by COST BM1405 and BM1408 EU actions.”

now reads:

“This work was supported by the National Science Centre Grant - UMO-2015/17/B/NZ3/00557 (J.W., M.R., M.M.) and by an EMMA-West grant from the EU (S.B.). S.B. was also supported by a FASTTRACK grant (SR/FTP/ETA-04/2012) from DST and a UGC Research Award (F.30-31/2016 SA-II) from the Government of India. M.M was also supported by the National Science Center Grant 2016/21/N/NZ3/03273. N.D. was supported by the PURSE-II project (Department of Science and Technology, Government of India). D.P. and S.B. were supported by the Polish National Science Centre (2014/15/B/ST6/05082), and by Foundation for Polish Science (TEAM to D.P.). D.P. was also supported by grant 1U54DK107967-01 ‘Nucleome Positioning System for Spatiotemporal Genome Organization and Regulation’ within 4DNucleome NIH program (TEAM grant to D.P.) and COST BM1405 and BM1408 EU actions.”

These errors have now been corrected in the PDF and HTML versions of the Article.

